# Quality of Life in Parkinson's Disease Caregivers: The Contribution of Personality Traits

**DOI:** 10.1155/2013/151872

**Published:** 2013-08-19

**Authors:** Eloise H. Tew, Sharon L. Naismith, Marilia Pereira, Simon J. G. Lewis

**Affiliations:** Parkinson's Disease Research Clinic, Brain and Mind Research Institute, University of Sydney, Sydney, NSW 2050, Australia

## Abstract

Parkinson's disease imposes significant demands not only on patients but also on those people living and caring for them, who often have a reduction in their quality of life. The factors that may ameliorate these effects, such as an individual's personality, are not understood. Therefore, the aim of this study was to look at the relative contribution of caregiver personality on their quality of life, specifically attempting to identify those traits, which may be protective or harmful. Two hundred and seventy-four caregivers of patients with Parkinson's disease were included in this study. Caregivers were given questionnaires to complete, including the Big Five Inventory and the World Health Organisation Quality of Life BREF version. Univariate correlations demonstrated that depression and anxiety were the largest predictors of reduced quality of life amongst caregivers. However, after controlling for these potential confounds, conscientiousness was associated with enhanced psychological quality of life and openness positively predicted benefits in the environmental domain. Neuroticism was associated with reduced quality of life in the psychological domain. Thus, screening for neuroticism may help identify those caregivers who would benefit from intervention strategies, which could in the long term help reduce the need for nursing home placement of Parkinson's disease patients.

## 1. Introduction

Parkinson's disease (PD) is the world's second most common neurodegenerative disease affecting 1%-2% of the population over 65 years of age [1]. It is a progressive condition with both motor and nonmotor symptoms that can have a profound impact not only on the patients but also on family members who often adopt the role of a caregiver [1].

PD can place significant demands on the caregiver, as they take on more daily tasks and increasingly provide physical, emotional, and economic support [2, 3]. It is recognised that caregivers of people with PD have a reduced quality of life (QoL) [4], where their social activities and work schedules reduce to be more involved in caring [5]. Caregiver spouses are less likely to spend time outside of the house or take a holiday than non-caregiver spouses [5], and older spousal caregivers often have to face age-related challenges themselves.

Preceding data has shown a relationship between advancing stages of PD and a caregiver's QoL, including an increase in strain and a negative lifestyle change as PD progresses [4]. This reduction in QoL can be related to a range of physical, psychological, and sociodemographic factors including the patient's age, disease stage and duration, cognitive and behavioural disorders associated with PD, years of caring, and level of education [2]. 

Caregiver burden (CB) is a factor that increases in caregivers looking after relatives with PD. CB refers to the stress and impact of looking after a relative and refers to the physical, mental, and socioeconomic problems that caregivers may encounter [6]. There is a significant correlation between increasing CB and a reduction in a caregiver's QoL [7] and level of depression [8]. Research has shown that PD caregivers have more psychological distress than the general population [9]. This commonly manifests with higher levels of depression, anxiety, and stress, which are associated with increasing disease progression [9].

Whilst a number of factors clearly impact on a PD caregiver's QoL, no known research has examined whether a caregiver's personality confers vulnerability to decreased QoL or conversely offers protection. Personality is an important consideration as it allows for a broader understanding of the caregiver's psychological profile. Whilst multiple personality traits have been proposed, there appear to be five key traits, namely extroversion, agreeableness, conscientiousness, openness, and neuroticism [10]. For further review, see Carver and Connor-Smith 2010 [11]. Extroversion describes someone who is confident, has lots of energy, and enjoys social interaction. Agreeableness refers to someone who is friendly, compassionate, and trusting. Conscientiousness refers to when a person is efficient, is organised, shows self-discipline, and aims for achievement. Openness, or being open to experience, is used to describe a person who is inventive, curious, and open emotionally. Finally, neuroticism defines someone who is sensitive and nervous and delivers unpleasant emotions easily.

Data has shown that personality characteristics appear to affect the processes people use to appraise stressful events and influence their ability to cope in the absence of caregivers [11]. Furthermore, personality factors relate significantly to QoL [12]. In particular, a number of studies have demonstrated the link between high levels of neuroticism and subsequent stress, decreased QoL, poorer health, and depression in non-caregivers [13–15]. It has been suggested that people expressing neuroticism need better coping methods and relaxation strategies, in order to decrease their psychological distress [11]. In contrast, a high level of extroversion in caregivers of patients with dementia appears to be protective, with data showing it is associated with decreased CB and depression, which may contribute to an improved QoL [16]. One recent study investigating familial caregivers of community-dwelling older adults with multiple functional impairments demonstrated that whilst extroversion was positively associated with better physical and mental health, neuroticism showed a negative relationship [17]. In addition, conscientiousness, openness, and agreeableness have all been positively associated with better QoL in studies of non-PD caregivers [14].

It has been recognised that caregivers play a pivotal role in preventing the transition of PD patients to nursing home care [18]. Therefore, identifying PD caregivers at highest risk of reduced QoL is of clinical relevance given that a number of factors, such as the frequency of caregiver breaks and perceived social support, can help to ameliorate the unfavourable effects of caregiving [19]. Such factors are also linked to psychological distress. Therefore, in the current study, we sought to identify the relative contribution of personality traits to various aspects of a PD caregiver's QoL, in the context of other influential factors such as mood.

## 2. Materials and Methods

### 2.1. Participants

This study assessed 274 caregivers of patients with PD. Caregivers were recruited from both rural (*n* = 100, 36.5%) and metropolitan areas (*n* = 174, 63.5%) across New South Wales, Australia. Caregivers included anyone who lived with a participant with PD, whether they were a spouse, relative, or friend.

All caregivers were asked to complete questions that were self-reported, which included demographic questions and psychological questionnaires. Patients were assessed by health professionals with specialist expertise in PD. All procedures were carried out with adequate understanding and written consent in all participants with ethical approval from the University of Sydney.

### 2.2. Caregiver Measures

#### 2.2.1. Personality

The Big Five Inventory-10 (BFI-10) [20] was used to evaluate the five types of personality. BFI-10 was chosen over the BFI-44 because of time constraints and length of the questionnaire pack given to the caregivers. The questionnaire consists of ten questions where two questions relate to each personality domain. The BFI-10 is scored from “Disagree Strongly” (1) to “Agree Strongly” (5) with the highest score indicating more of that personality trait (score range for each domain = 2–10). Some of the five personality factors are measured using a reversed scoring system.

#### 2.2.2. Quality of Life

The World Health Organisation Quality of Life Scale BREF version (WHOQoL-BREF) [21] contains 26 questions measuring QoL using a 5-point scale ranging from “Very Dissatisfied” to “Very Satisfied.” There are four domains that can be calculated from the questionnaire; “Physical Health,” “Psychological Health,” “Social Relationships,” and “Environment”. The transformed scores from the raw data are ranged from 0 to 100, with the higher scores indicating a better QoL. The internal consistency, measured by Cronbach's *α*, is satisfactory to good (*α* > 0.70) [22]. Where >20% of data was absent from a caregiver's questionnaire, that subject's data was considered missing. Where >2 items were missing from a domain, that domain score was not calculated (exception of the social relationship domain, where it was calculated if <1 item is missing) [21].

#### 2.2.3. Anxiety and Depression

Psychological distress was measured using the Hospital Anxiety and Depression Scale (HADS) [23], which contains 14 items grouped into two domains: anxiety and depression. The questionnaire is rated on a 4-point response from 0 to 3 with “0” indicating no psychological distress at all and “3” indicating worse psychological distress. Anxiety and depression were measured individually. Where >5% of answers were absent from a domain, scores were considered missing for that domain.

#### 2.2.4. Demographics

Caregiver demographics included age, gender, marital status, relationship with PD patient (spouse, partner, child, or sibling), employment (employed, unemployed, retired/pensioner, or caregiver), education (Pre year 10, secondary school certificate, technical and further education (TAFE), and university), domestic services required, and any significant caregiver medical and/or mental health concerns. Demographics also included questions about caring for their companion with PD: duration of care (years), whether they received payment for care, what time of the day they help with caregiving (day, night, or both), and the number of hours per day they assist with activities of daily living (ADLs) such as showering, feeding, toileting, assistance with ambulation or transferring, dressing, and grooming.

### 2.3. Patient Measures

The physical stage of PD was measured using the Hoehn and Yahr rating scale (H&Y) [24] and other details including age, gender, marital status, and disease duration (years since diagnosis) were recorded.

### 2.4. Statistical Analysis

All statistics were generated using the Statistical Package for the Social Sciences (SPSS) version 20.0. Univariate correlations were performed for continuous variables evaluating the relationship between the QoL domains, personality traits, and other contributing factors using Pearson's correlation. Four stepwise linear multiple regressions were then constructed based on significant univariate correlations, with forced entry of key potential confounds in block 1 and personality variables entered into block 2 (method = enter) and the final full model with standardised beta (b) weights and adjusted *R* ^2^ reported. Analyses were two tailed, with an alpha of 0.05. Correlation strengths were rated as weak (0.2–0.39), moderate (0.4–0.59), and strong (>0.6).

## 3. Results

As shown in Table 1, 65% of caregivers were females with their spouse/partner as their significant other with PD. The mean age of caregivers was 65 years old and they had been caring for the patient for around four years. Just over half were retired and 29.6% of caregivers continued their education at University. Participants with PD were slightly older (69.9 years ±9.6) and had a disease duration of 7.9 (±6.2) years with an average H&Y score of 2.5.

### 3.1. Univariate Relationships between QoL and Personality

As shown in Table 2, in the physical health domain, lower levels of caregiver QoL were correlated with greater patient disease duration, advanced stage of disease, older age of the caregiver, greater duration of care, more hours spent assisting with ADLs, higher caregiver neuroticism, anxiety, and depression. Conversely, better physical QoL in caregivers was correlated with higher levels of conscientiousness and openness.

Lower levels of caregiver psychological QoL were associated with greater patient disease duration, advanced stage of disease, longer duration of caring, more hours spent assisting with ADLs, and higher levels of caregiver neuroticism, anxiety, and depression. In contrast, better psychological QoL amongst caregivers was associated with higher levels of conscientiousness and extroversion.

In the social relationship domain, reduced caregiver QoL was associated with advanced stage of disease, longer duration of caring, more hours spent assisting with ADLs, higher levels of caregiver neuroticism, anxiety, and depression. In addition, higher social relationship QoL was associated with higher levels of caregiver conscientiousness.

Lower levels of the environmental QoL were associated with advanced stage of disease, longer duration of caring, more hours spent assisting with ADLs, higher levels of caregiver neuroticism, anxiety, and depression. On the other hand, a better environment QoL score for the caregiver was associated with greater levels of openness.

There were no significant associations between any QoL domain and the personality trait of agreeableness.

### 3.2. Multivariate Analyses

Four multiple linear regression models were constructed with forced entry of all univariate predictors (as determined above) in block 1, and personality variables entered in block 2.

When controlling for the effects of patient disease duration, disease stage, caregiver age, depression, anxiety and hours assisting with ADLs for the physical health, and social relationship QoL domains, personality was no longer a significant predictor. The only significant predictor was depression (*b* = − 0.39, *P* < 0.001) in the social relationship domain (full model *F*_7,223_ = 10.9, *P* < 0.001, adjusted *R* ^2^ = 23.1%).

Both neuroticism (*b* = − 0.14, *t* = − 2.6, *P* < 0.05) and conscientiousness (*b* = 0.10, *t* = 2.0, *P* < 0.05) remained significant predictors of psychological QoL even after controlling for the patient disease stage (*b* = 0.01, *t* = 0.1, ns), disease duration (*b* = −0.11, *t* = −1.8, ns), duration of caregiving (*b* = 0.01, *t* = 0.2, ns), number of hours spent assisting with ADLs (*b* = −0.02, *t* = −0.4, ns), depression (*b* = −0.31, *t* = − 3.9, *P* < 0.001), and anxiety (*b* = −0.31, *t* = −3.9, *P* < 0.001). Overall, this combination of variables predicted 48.0% (adjusted *R* ^2^) of variance in psychological QoL (full model *F*_9,221_ = 24.6, *P* < 0.001). In contrast, extroversion was no longer a significant predictor after accounting for these other factors.

For the caregiver's environmental QoL, even after controlling for disease stage (*b* = 0.02, ns), duration of caregiving (*b* = −0.02, ns), hours spent assisting with ADLs (*b* = 0.20, *P* < 0.001), depression (*b* = −0.40, *P* < 0.001), and anxiety (*t* = −0.19, *P* < 0.001), the personality trait of openness remained a significant predictor (*b* = 0.13, *P* < 0.01), though neuroticism did not. Overall, these variables explained 44.5% (adjusted *R* ^2^) of variance in environmental QoL (full model *F*_7,223_ = 27.4, *P* < 0.001).

## 4. Discussion

This is the first study to examine the relative contributions of personality to QoL in caregivers of PD patients. The main findings of this study confirm that depression and anxiety represent the largest negative predictors of QoL in caregivers and additionally highlight the role of personality factors. Neuroticism was negatively associated with psychological QoL amongst caregivers whereas conscientiousness, openness, and extroversion appeared to be positively protective. This large sample of PD patients and caregivers allowed multivariate modelling of personality attributes whilst controlling for possible confounds related to the patient and the caregiver. These analyses showed that personality variables did not predict QoL in social or physical domains, once all other confounds were considered.

Lower QoL in the psychological domain amongst caregivers was associated with being more neurotic. This suggests that caregivers who show a high level of neuroticism need increased levels of psychological support, as they may struggle to cope with stressful situations and an over-bearing load of work. The psychological domain of QoL was associated with depression and anxiety emphasising the fact that these symptoms may be compounded in caregivers with increased rates of neuroticism. This highlights the need for an awareness of this combination of personality type and affective disorder. Such relationships have been highlighted in the spouse-caregivers of patients with Alzheimer's disease [25]. This work has identified a differential improvement in depression that was related to levels of neuroticism, suggesting that there is an opportunity to target psychological interventions for caregivers with lower levels of neuroticism [25]. A recent study evaluating a targeted intervention program for PD patients and their caregivers showed a beneficial effect on mood in both groups that lessened the psychosocial problems of the caregiver [26]. However, that study did not include a measurement of the caregiver's personality, which suggests that in clinical practice, incorporating screening of personality and psychological distress may be worthwhile to detect those caregivers for intervention programs early.

Better QoL in the psychological domain was associated with caregivers being more conscientious. Conscientious people have been shown to have problem-solving coping mechanism with strong attention regulation, which allows them to disengage from negative thoughts allowing them to have a better psychological health [11]. This coping method could be beneficial in caregivers with high levels of neuroticism [16, 27]. A person who is conscientious may overcome unexpected obstacles more easily than a person who is less motivated to achieve important life tasks [12]. In this context, such people may gain a sense of pleasure and gratification from successfully operating in the role of a caregiver.

Poorer QoL in the environmental domain of the caregivers was associated with the hours they spent helping with ADLs, depression, and anxiety levels. This domain reflects issues relating to financial burden, concerns about physical safety, health and social care, and the home environment. Many of these concerns are very difficult to overcome but potentially could be improved by an increased knowledge and awareness about PD and the services available to support caregivers and patients in the rural or metropolitan areas [28]. It is hoped that such information could be successfully coordinated by having greater access to specialist PD nurses [29, 30]. Interestingly, openness had a positive effect on the environmental domain of QoL amongst caregivers. This might reflect the fact that those caregivers having the openness personality trait tend to ask more questions and are amenable to new experiences, adapting to potential stressors.

One possible limitation of this study would be its reliance on self-reported questionnaires and it is feasible that clinical interview may have offered more detailed insights. However, these tools have all been well validated in previous work. Furthermore, the responses in a clinical interview might be more guarded by caregivers being concerned about judgment. Another limitation is that the briefer BFI-10 was used instead of the more in depth BFI-44, which may have lost some reliability, especially for the personality trait of agreeableness [20]. However, there were clear time constraints on the caregiver and a necessity to avoid overloading them. Future studies may seek to address this limitation by use of more in-depth measures of personality. In terms of data interpretation, it is also possible that this study has an inflated type I error rate, since we did not apply a Bonferroni correction to the alpha levels used. Since this study was the first of its kind, we opted to leave the alpha level at 0.05 but acknowledge that future studies may wish to be more stringent in this regard.

Overall, this study supports that personality traits play a crucial role in the QoL of PD caregivers. Our results highlight that neuroticism negatively impacts on psychological well-being, even after controlling for other potential confounds. In contrast, psychological QoL was improved in those caregivers with higher levels of conscientiousness. A personality trait with more openness improves the QoL relating to the environment of the caregiver. It is hoped that a greater appreciation of these effects may help health professionals to better support these individuals and in turn support those patients living with PD.

## Figures and Tables

**Table 1:** Carer and patient demographic data.

Demographics	Caregiver		Patients with PD	
	*n *	%	*n *	%
Region				
Metropolitan	174	63.5	174	63.5
Rural	100	36.5	100	36.5
Gender				
Female	177	64.6	103	37.6
Male	84	30.7	171	62.4
Marital status				
Never married	11	4.0	4	1.5
Married/de facto	242	88.3	241	88.0
Separated	8	2.9	4	1.5
Widowed	0	0.0	16	5.8
Divorced	0	0.0	9	3.3
Relationship				
Spouse/partner	227	82.8		
Child	25	9.1		
Sibling	3	1.1		
Friend	2	0.7		
Other	4	1.5		
Employment				
Employed	72	26.3		
Unemployed	2	0.7		
Retired/pensioner	152	55.5		
Carer	24	8.8		
Other	10	3.6		
Education				
No formal education	2	0.7		
Preyear 10	53	19.3		
Secondary school certificate	59	21.5		
TAFE	60	21.9		
University	81	29.6		
Other	5	1.8		

TAFE: Technical and further education; PD: Parkinson's disease.

**Table 2:** Univariate relationships between carer quality of life and personality.

	Caregiver WHOQoL-BREF			
	Physical health	Psychological health	Social relationship	Environment
Patient				
Disease duration	−0.17**	−0.17**	−0.11	−0.12
Age	−0.10	−0.02	−0.02	−0.04
Hoehn and Yahr [24]	−0.18**	−0.20**	−0.18**	−0.23**
Caregiver				
Age	−0.15*	0.01	−0.02	−0.04
Duration of caregiving	−0.32**	−0.20**	−0.21**	−0.26**
Hours per day assisting with ADL	−0.22**	−0.15*	−0.17**	−0.34**
Neuroticism	−0.29**	−0.44**	−0.25**	−0.28**
Extroversion	0.09	0.20**	0.10	0.11
Agreeableness	−0.10	0.02	0.10	−0.03
Conscientiousness	0.17**	0.21**	0.14*	0.11
Openness	0.13*	0.11	0.08	0.18**
Anxiety	−0.44**	−0.63**	−0.43**	−0.57**
Depression	−0.53**	−0.63**	−0.47**	−0.62**

*r* values above are significant at *P < 0.05 level (2-tailed) and **P < 0.01 level (2-tailed). ADL: activities of daily living; WHOQoL-BREF: World Health Organization Quality Of Life-BREF version.
